# Clinical Course of Hyperprolactinemia in Children and Adolescents: A Review of 21 Cases

**DOI:** 10.4274/jcrpe.v3i2.14

**Published:** 2011-06-08

**Authors:** Erdal Eren, Şenay Yapıcı, Esra Deniz Papatya Çakır, Latife Aytekin Ceylan, Halil Sağlam, Ömer Tarım

**Affiliations:** 1 Department of Pediatric Endocrinology, Uludağ University, Faculty of Medicine, Bursa, Turkey; 2 Department of Pediatrics, Uludağ University, Faculty of Medicine, Bursa, Turkey

**Keywords:** Prolactin, puberty, prolactinoma, Pituitary

## Abstract

**Objective:** Hyperprolactinemia may be due to various etiological factors and may present with different signs and symptoms. It is relatively less frequent in childhood than in adulthood.  The aim of this study was to evaluate retrospectively the clinical course and outcome of hyperprolactinemia in pediatric patients.

**Methods:** We investigated the records of 21 patients with hyperprolactinemia who attended a tertiary hospital.

**Results:** Menstrual problems, galactorrhea , and headache  were the most common presenting symptoms. Hyperprolactinemia was due to microadenoma in 10, macroadenoma in 7, and was drug-induced in 4 patients. Bromocriptine and cabergoline were equally effective in lowering serum prolactin levels.  Surgical treatment in children with macroprolactinoma was not curative and dopamine agonist therapy was required postoperatively.

**Conclusion:** In the presence of any clinical symptom or sign suggestive of suppression of the pituitary-gonadal axis, hyperprolactinemia should not be forgotten as a probable diagnosis. Medical therapy seems effective in microadenoma. Surgical therapy may not be successful in macroadenoma and recurrence is frequent.

**Conflict of interest:**None declared.

## INTRODUCTION

A variety of etiological factors including disorders of the hypothalamo-pituitary axis, interruption of dopamine synthesis, stress, pituitary tumors, polycystic ovary syndrome, primary hypothyroidism, and various medications may lead to hyperprolactinemia (1). Hyperprolactinemia in girls causes delayed puberty, hypogonadotropic hypogonadism, primary or secondary amenorrhea, and galactorrhea (2). The clinical åpicture in boys includes delayed puberty, gynecomastia, and galactorrhea as well as neuro-ophthalmologic findings such as impaired vision and headache due to a higher frequency of macroadenomas (3). Prolactinoma is the most common hormonally active pituitary adenoma which usually presents in adulthood (4). Therefore, epidemiologic and clinical data in children are limited. In this investigation, we aimed to determine the etiology, clinical findings, and management of hyperprolactinemia in children and adolescents at a tertiary hospital. 

## MATERIALS AND METHODS

A total of 21 patients with hyperprolactinemia, followed in Uludağ University Division of Pediatric Endocrinology between July 2006 and July 2010, were studied retrospectively. The presenting symptoms, associated diseases, medications, physical and laboratory findings were recorded. The serum levels of prolactin, follicle-stimulating hormone (FSH), luteinizing hormone (LH), estradiol (E2), testosterone (T), thyrotropin (TSH), and free thyroxine (fT4) were measured by the Architect device using chemiluminescent microparticle enzyme immunoassay. A prolactin level of 5-20 ng/mL was considered normal in both sexes. A level above 20 ng/mL in two successive measurements was defined as hyperprolactinemia (5). Magnetic esonance imaging (MRI) of the pituitary gland was performed in all patients. A pituitary adenoma with a diameter of less than 1 cm was defined as microadenoma and one above 1 cm in diameter as macroadenoma. Patients with macroadenoma underwent transsphenoidal pituitary surgery. Medical treatment was given to the subjects with microadenoma, persistent postoperative hyperprolactinemia, and to those with hyperprolactinemia due to medications. Bromocriptine 2.5 mg (Parlodel®, Novartis) once or twice a day or cabergoline 0.5 mg (Dostinex®, Pharmacia) once or twice a week was given as prolactin-lowering drug. Bromocriptine or cabergoline was selected randomly and according to the availability of the medicine in the market. Serum prolactin levels were monitored at 2-4 weeks after the initiation of treatment and 3-6 months thereafter. Mann-Whitney U test was used to compare groups for continuous variables, and Fisher’s exact test was used for categorical variables. A p value of less than 0.05 was considered significant. SPSS 16.0 statistics program was used for analysis.  

## RESULTS

A total of 21 patients [17 girls (81%) and 4 boys (19%)] with hyperprolactinemia were included in the study. Mean age at diagnosis and anthropometric data are presented in Table 1. The presenting symptoms in the female patients were irregular menstruation in 9, galactorrhea in 6, headache in 3, and primary amenorrhea in 4. Three patients were asymptomatic, two of whom were receiving antipsychotic medication. Among males, headache was the presenting symptom in 2, gynecomastia in 1, galactorrhea in 1, and blurred vision in 1. One patient was diagnosed based on an elevated prolactin level in a random blood sample and turned out to have a macroadenoma. None of the patients had papilledema at fundoscopic examination. Mean prolactin levels are summarized in Table 2 and the clinical course and prolactin levels of individual patients are given in detail in Table 3a and 3b.

The initial serum levels of FSH, LH, E2, T, TSH, and fT4 were within normal ranges and did not show any significant changes after treatment (data not presented). All male patients had macroadenoma and underwent surgical resection. None of the patients had post-surgical visual problems. Prolactinoma was histopathologically confirmed in all surgical specimens. The serum prolactin level was significantly higher in patients with macroprolactinoma than in those with microadenoma (p=0.024). Patients with hyperprolactinemia due to antipsychotic medication tended to have lower levels of prolactin compared to those with microadenoma, but the difference was not significant. The serum prolactin level in 2 of the 4 patients in this group decreased to normal after cessation of the antipsychotic medication. 

One of the other 2 patients with persistent hyperprolactinemia had microadenoma on MRI, while the second one had normal MRI. Prolactin-lowering medication was continued in both of these patients. MRI revealed microadenoma in 7, macroadenoma in 7, ‘partially empty sella’ in 1, and normal pituitary gland in 1 patient. Six of the 7 patients with macroadenoma who had pituitary surgery required medical treatment for persistent postoperative hyperprolactinemia. 

**Figure 1:**
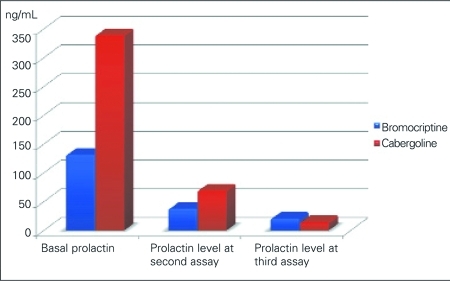
Comparison of effect of prolactin-reducing medications

Bromocriptine was given to 13 (52%) patients and cabergoline to 6 patients (23%) as medical treatment. The initial prolactin level was not significantly different between the two groups (p=0.257). The progression of prolactin level in the two treatment groups is demonstrated in Figure 1. Both therapies seemed to be equally efficacious. No significant side effects were observed, except for mild nausea and drowsiness. 

**Table 1:**
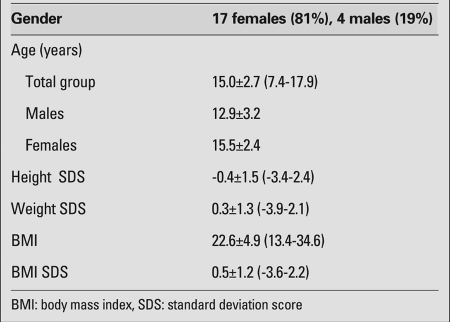
Auxological data of the hyperprolactinemia patients  (mean values)

**Table 2:**
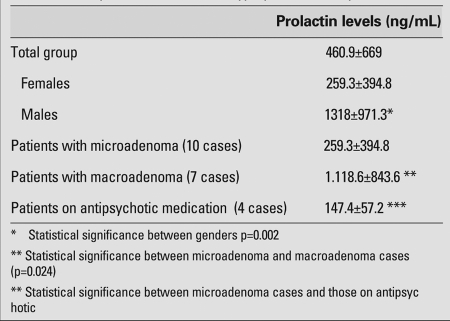
Mean prolactin levels of the hyperprolactinemia patients

**Table 3:**
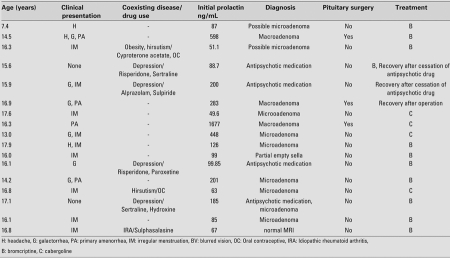
Age, diagnosis, clinical presentation, prolactin level, and treatment modes of  female hyperprolactinemia patients

**3:**
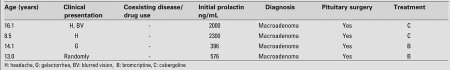
Age, diagnosis, clinical presentation, prolactin level, and treatment modes of  male hyperprolactinemia patients

## DISCUSSION

Although prolactinoma is the most frequent pituitary adenoma, it is relatively rare in childhood. It usually presents with menstrual problems late in childhood, in adolescent years (6). The clinical signs and symptoms vary according to age, sex, tumor size, and prolactin level. Headache, amenorrhea, and galactorrhea are reported as the major presenting symptoms. Headache is more common in males (67-77% vs. 17-30% in females) and does not seem to be related to the tumor size or prolactin level (7). Seven patients with macroadenoma in our study (all of the 4 male patients and 3 girls) presented with headache. None of the patients had visual problems, except for one who complained of blurred vision that was attributed to myopia. Menstrual irregularity and/or amenorrhea were observed in 76.5% of our female patients, a finding which was consistent with the literature. Fideleff et al (2) have reported primary amenorrhea in 29-45% and oligomenorrhea in more than 29% of their patients. Four of our patients with primary amenorrhea started menstruating after therapy. Galactorrhea has been reported in 30-50% of the girls with prolactinoma (2,6). Among our patients, 41.2% (6 girls, 1 boy who also had gynecomastia) had galactorrhea. Saranac et al (8) have studied 11 children with hyperprolactinemia and reported short stature and obesity in 4. The authors recommended measurement of prolactin levels in short and obese children. In contrast, only 2 of our patients had a height for age below-2SD and only 1 had a BMI value exceeding +2SD. 

A number of drugs used in psychiatric clinics lead to  an increase in serum prolactin levels. This effect is dose-dependent and the elevated serum prolactin returns to normal after the responsible drug is discontinued (9,10).  Drug-induced hyperprolactinemia is usually mild (<200 ng/mL) (5). Among our patients, 4 had drug-induced hyperprolactinemia and one of these patients was also diagnosed to have microadenoma. 

Dopamine agonists are the drugs of choice for treatment of micro- and macroadenomas. Bromocriptine, cabergoline, pergolide, and quinagolide inhibit prolactin secretion by exerting G-protein-mediated D2 dopamine agonist effect. Bromocriptine suppresses pituitary mitosis and induces apoptosis and perivascular fibrosis, which lead to diminished tumor size. Bromocriptine is administered twice a day and is notorious for its side effects like nausea, vomiting, postural hypotension, and mental dullness (11). However, our patients tolerated bromocriptine well with no major side effects. Increasing the dose slowly appears to increase tolerability. Cabergoline has an advantage of less frequent dosing and it is reported to be equally effective (6). We also observed that both drugs were efficacious in lowering the prolactin level and did not lead to any significant side effects. 

Dopamine agonist therapy per se has been reported to be adequate for macroprolactinoma in adults (12,13,14). However, experience in children is limited. Acharya et al (18) have studied 39 children with hyperprolactinemia including 14 with macroadenoma with suprasellar extension. Dopamine agonist therapy was required in all of the patients postoperatively. Six of the 7 patients with macroadenoma in our study also required dopamine agonist therapy after surgical resection. This finding is in contrast to the remission rates as high as 53-67% which have been reported in adult studies (15,16,17). However, postoperative hypopituitarism also has been more frequently found in adult studies (13,14,18). No pituitary insufficiency was observed in our patients after surgery. This may suggest that curative surgery requires more extensive resection. However, more extensive resection may also lead to higher rates of postoperative hypopituitarism. In adults, medical therapy is recommended even in patients with a macroadenoma and even in the presence of visual field pathology (19). In children and adolescents, the guidelines are not well defined. We chose to refer the patients for surgery in the presence of macroadenoma. We do not know what the results would have been if medical therapy was initiated. However, we know that surgical therapy alone does not cure and postoperative medical therapy is needed.

In conclusion, patients with hyperprolactinemia, including those with prolactinoma, may present with different symptoms.  Any clinical symptom or sign that suggests suppression of the pituitary-gonadal axis must be taken as a reminder for consideration of hyperprolactinemia as a probable diagnosis. Drug-induced hyperprolactinemia may need to be treated with dopamine agonists, but prolactinoma must be excluded. Surgical treatment in children with macroprolactinoma is usually not curative and dopamine agonists must be continued postoperatively. Medical therapy may be recommended as first-line treatment for micro-and macroadenoma, but further studies are needed in children.
